# Preventive Evidence into Practice: what factors matter in a facilitation intervention to prevent vascular disease in family practice?

**DOI:** 10.1186/s12875-019-0995-7

**Published:** 2019-08-08

**Authors:** Grant Russell, Riki Lane, Sharon Parker, John Litt, Danielle Mazza, Jane Lloyd, Nicholas Zwar, Mieke van Driel, Chris Del Mar, Jane Smith, Mark F. Harris, On behalf of the Preventive Evidence into Practice (PEP) Partnership Group, Mark Harris, Nicholas Zwar, John Litt, Danielle Mazza, Mieke van Driel, Richard Taylor, Grant Russell, Chris Del Mar, Jane Lloyd, Jane Smith, Elizabeth Denney-Wilson, Gawaine Powell Davies, Rachel Laws, Teri Snowdon, Helen Bolger-Harris, Stephan Groombridge, Stan Goldstein, Teresa Howarth, Nancy Huang, Jinty Wilson

**Affiliations:** 1 Southern Academic Primary Care Research Unit, Department of General Practice, Monash University, Building 1, 270 Ferntree Gully Road, Notting Hill, Vic 3168 Australia; 2 Centre for Primary Health Care and Equity, University of New South Wales, Sydney, NSW 2052 Australia; 3 Discipline of General Practice, Health Sciences Building, Flinders University, Adelaide, SA 5042 Australia; 4 Department of General Practice, Monash University, Building 1, 270 Ferntree Gully Road, Notting Hill, Vic 3168 Australia; 5 School of Public Health and Community Medicine, University of New South Wales, Kensington, NSW 2052 Australia; 6 Primary Care Clinical Unit, Faculty of Medicine, University of Queensland, Brisbane, QLD 4029 Australia; 7 Faculty of Health Sciences and Medicine, Bond University, Gold Coast, QLD 4229 Australia

**Keywords:** Primary care, Family medicine, Outreach facilitation, Preventive care, Qualitative research

## Abstract

**Background:**

A perennial challenge of primary care quality improvement is to establish why interventions work in some circumstances, but not others. This study aimed to identify factors explaining variations in the impact on clinical practice of a facilitation led vascular health intervention in Australian family practice.

**Methods:**

Our mixed methods study was embedded within a cluster randomised controlled trial of a facilitation intervention designed to increase the uptake of evidence-based prevention of vascular disease in family practices. The study was set in four Australian states using eight of the study’s 16 intervention practices. Facilitators worked with intervention practices to develop and implement improvements in preventive care informed by a vascular risk factor audit. We constructed case studies of each practice’s “intervention narrative” from semi-structured interviews with clinicians, facilitators and other staff, practice observation, and document analysis of facilitator diaries. The intervention narratives were combined with pre- and post-intervention audit data to generate typologies of practice responses to the intervention.

**Results:**

We found substantial variability between practices in the changes made to vascular risk recording. Context (i.e. practice size), adaptive reserve (i.e. interpersonal relationships, manager and nurse involvement), and occasional data idiosyncrasies interacted to influence this variability.

**Conclusion:**

The findings emphasise the importance of tailoring facilitation interventions to practice size, clinician engagement and, critically, the organisation of, and relationships between, the members of the practice team.

**Trial registration:**

The trial was registered with the Australian and New Zealand Clinical Trials Registry (ANZCTR): ACTRN12612000578808 (29/5/2012). This trial registration is retrospective as our first patient returned their consent on the 21/5/2012. Patient recruitment was ongoing until 31/10/2012.

**Electronic supplementary material:**

The online version of this article (10.1186/s12875-019-0995-7) contains supplementary material, which is available to authorized users.

## Background

Primary care has a key role in the prevention of chronic disease [1, 2], particularly that affecting the cardiovascular system. Evidence is accumulating as to the value of outreach facilitation in improving the primary care practice based prevention of cardiovascular disease (CVD) [3].

Outreach facilitation represents a *“strategy to improve … processes and outcomes, including the delivery of wellness and preventive services, through the creation of an ongoing, trusting relationship between an external facilitator and a primary care practice.”* In the last decade, more has become known about the optimal characteristics of successful facilitation programs [3, 4]. However, despite increasing evidence of the relationship between context and the success of primary care reform initiatives [5–8], little is known about the qualities of a primary care practice that make it more likely to respond to a facilitation intervention [9].

*Preventive Evidence into Practice* (PEP) [10] was a cluster randomised control trial of outreach facilitation to support the prevention of CVD in patients aged 40 to 69 years, in 30 general practices in four Australian States. PEP’s outreach facilitation intervention (Additional file 1) was designed to achieve improvements in 1) the documentation of behavioural and physiological risk factors, and 2) adherence to guidelines for the management of these risk factors. While PEP intervention practices made modest improvements in the recording of behavioural risk factors such as alcohol consumption, smoking, waist circumference, and body mass index (BMI), the intervention did not influence intermediate patient outcomes such as BMI, waist circumference, and blood pressure (BP). While a regression analysis suggested that smaller practices and those with two or more nurses were more likely to improve assessment and recording of BP, substantial and unexplained inter-practice variability remained [10].

We used a mixed methods approach to identify organisational factors explaining variations in the impact of a facilitation led vascular health intervention delivered to a sample of Australian primary care practices.

## Methods

Our explanatory mixed methods study [11] used case studies of general practices participating in the intervention arm of PEP. Intervention narratives were created using qualitative data to explain quantitative practice-based CVD preventive performance at baseline and in response to the intervention. Case study approaches have an increasing role in health services research, particularly when the unit of analysis is an organisation [12]. They involve collection of qualitative (and often quantitative) data from various sources to explore organisations and the characteristics of their contexts [13]. We used a framework grounded in complexity theory and complex adaptive systems [14]. Our exploration of the contextual influences of uptake of the PEP intervention drew on Stange and Glasgow’s conceptual model of the influence of context on primary care transformation [5].

Recruitment for the PEP study is described elsewhere [10]. Our sample of eight general practices was drawn from PEP’s 15 intervention practices using a maximum variation sample of the study practice selection criteria (practice size and location) to select two practices from each participating Australian state (New South Wales, Victoria, Queensland, and South Australia). Participants included practice intervention facilitators, practice-based staff (general practitioners, practice nurses (PNs), and practice managers (PMs). There were five facilitators (two in Queensland and one in each of the other states); four had prior nursing qualifications and had worked either with or within general practices in the past.

### Qualitative data

Data was collected by semi-structured face to face and/or telephone interviews with participants. Practice staff were interviewed twice: first, after the facilitators had completed at least one practice visit; and subsequently at completion of the intervention. Facilitators were interviewed following the intervention. Both sets of interviews followed interview guides. Staff interview guides (Additional file 2) were modified from a design used in an earlier study examining the effect of external facilitators in enhancing the delivery of chronic condition care planning in Ontario primary care [15]. Facilitator interview guides (Additional file 3) were further informed following initial analysis of the practice interviews and facilitator reports. Both guides were progressively modified following the iterative process of data collection and analysis.

Early staff interviews provided an understanding of the clinicians’ and, where possible, the practice’s approach to guideline related preventive care of CVD, in particular with respect to socially disadvantaged groups. Later interviews ascertained practice experiences with the implementation of PEP, particularly in relation to any changes in individual or practice-based routines linked with preventive care. Facilitators were asked to reflect on their perceptions of critical practice and practitioner factors influencing the fidelity of the intervention. Facilitator interview guides were developed based on initial analysis of the practice interviews and facilitator reports, then progressively modified with the iterative process of data collection and analysis.

The facilitators also compiled a) a practice profile template to capture reflective notes during the study and a report at the end of the intervention to describe their observations of preventive activities within the practice; and b) practice visit reports of each practice encounter. Facilitators used these reports to document practice goals, relevant activities, and perceived challenges and barriers to change within the practice.

State-based field research officers (all with post-graduate qualifications in health sciences), conducted practice staff interviews and observations. Facilitator interviews were conducted by a PhD social scientist (RL), with significant experience in qualitative interviewing and practice based primary care research. Each interview was audio-recorded and transcribed verbatim.

### Quantitative practice performance data

We used a clinical audit software to extract de-identified clinical data relating to the targets of the intervention from each participating site. Data was extracted at baseline and again, 12 months later. The practice was the unit of analysis. Data provided information on the extent of recording of risk factors for BMI, waist circumference, BP, alcohol intake, and smoking for patients aged 40–69 years; and lipids, fasting blood sugar level (BSL), and absolute CVD risk for patients aged 45–69 years.

Data management and analysis used two teams: one analysed qualitative data for each site, and another generated quantitative reports of each practice’s pre- and post-intervention performance.

The qualitative analysis involved coding of transcripts, field notes, and practice profiles using NVivo software [16] to generate “intervention narratives” of each practice’s experiences with the intervention. Next, we used an immersion crystallisation approach involving repeated cycles of detailed examination of sections of the data, followed by reflection, identification, and articulation of themes [17]. We then developed matrices comparing the practices across domains including: outer and inner context (physical space, provider and administrative roles and relationships); personnel; changes in the last year; priorities for prevention, information technology) and specific practice and facilitator experience of the intervention*.*

The quantitative analysis assessed pre-intervention documentation of the proportion of charts with sufficient data for the calculation of CVD risk (BP, smoking, serum cholesterol, and of the presence or absence of diabetes). Practices were ranked by the extent of baseline documentation of CVD risk (from practice A to H). We then measured: a) the proportion of records with data on BP, smoking, serum cholesterol, body mass index, waist circumference, BSL, alcohol consumption, and of the presence or absence of diabetes; b) the change in the proportion of records for which data was available for each variable at 12 months after the intervention; c) the change in the proportion of total eligible records that documented such change. Eligible records were those of patients fitting the enrolment criteria, but where documentation was not present at baseline. For example, if there were 1000 patient records in a practice and 60% had appropriate documentation for a risk factor (600 charts), then 400 charts could change. If at follow up 700 (70%) had appropriate documentation, the % that changed of eligible charts (those that could change) would be 100/400 charts (25%).

We highlighted differences of ≥10% and ≥ 20% from the pre- to post-intervention recording outcomes in both calculations. The broader PEP study was powered to identify changes of 20% recording of behavioural and physiological risk factors as a consequence of the intervention.

Finally, we compared quantitative and qualitative findings to identify principal explanatory factors by considering practice narratives alongside pre- and post-intervention audit data on varied dimensions of quality of preventive care. Initially separate teams analysed the quantitative and qualitative data sets. Then, both teams examined the qualitative data and discussed the narratives emerging from the data. We used a case study approach (where the case was the practice) to explore the mechanisms generating the characteristics and variability of baseline and intervention performance.

## Results

The eight practices ranged in size from 1 to 13 Full Time Equivalent (FTE) general practitioners. Two practices were in semi-rural areas and the remainder in outer suburbs of capital cities. Each had fully computerised medical records, a PM, and one to three PNs. Table 1 shows key characteristics of the practices, and Table 2 shows the changes in CVD preventive practice performance from baseline to 12 months following the intervention.

**Table 1:** Practice details and contextual levers

	Outer context^a^				Inner context				
					Practice core		Adaptive reserve		Attitude to intervention
	Relevant historical factors or recent events	Particulars of patient populations	Other external contextual issues i.e. rural setting	Links with the external environment	(i.e. Staffing IT maturity, staff roles and space)	Facilitative leadership	Aligned management model	Healthy relationship infrastructure	
A	Worked with AUSDRISK diabetes tool: mixed success.	High socio-economic status (SES), some migrants but “high health literacy”	Lack financial support for longer consults	Accreditation context for prevention. Medicare Local training on PEN-CAT software	Good 3 GPs. Inconsistent data entry	Strong PM	Aligned, whole of practice systems prevention focus prior	Good strong team	Very engaged – all clinicians participated. No prior facilitation experience
B	Stable practice	Mixed SES	Semi-rural practice. Few local specialists bulk bill	Good connections to allied health providers (AHPs), long distance to medical specialists	Good 1–2 GPs.	Strong	Partially aligned, through risk assessment and recall system	Strong	Organised and committed. All clinicians participated
C	Acted as a diabetes collaborative.	Medium/mixed SES	Suburban practice	Some visiting AHPs; can be cost barriers	Fair. Few systems. 13 GPs Very busy	PM felt let down by GPs	Partially aligned, variation for weight, height, alcohol, smoking	Fair – many meetings	Poor: 3/13 GPs participated
D	Long interest in HIV care	Medium-Low SES. Many of a non-English-speaking background, overseas students.	Suburban practice	AHP referrals for more difficult patients	Staff turnover during intervention, 4 GPs Inconsistent data entry	Hierarchic – leaders positive	Aligned roles post intervention Systems for PNs to see clients before GP	Dysfunctional staff tensions. PN resignation led to redeveloping a prevention team.	Lead GP and PM support. All GPs participated, but at varying levels Key PN opposition
E	A university teaching practice	Medium	Rural - People have to drive for services.	Good AHP connections at low cost	Good 2–3 GPs Good recall system	Strong	Whole of practice approach	Vibrant culture well organised and enthusiastic	Engaged – all clinicians participated
F	Utilise health check MBS items	High SES, mostly Caucasian employed families.	Suburban practice	Free gym passes	12 GPs Inconsistent data entry Cramped	Fair	Fragmented Nurse hired as prevention coordinator	Teamwork mostly informal. PNs overworked	Weakly engaged while PN champion on leave. Then good 5/8 GPs fully participated, 2 partly
G	New building new IT system	Low SES	Most clinical staff related to each other. Specialists’ cost an issue	AHP links	5+ GPs Poor – major IT change Cramped	PM led, but away for much of intervention	Disorganised PNs not at meetings	Fair Poor communications	Unresponsive 4/5 GPs weakly participated
H	Recently opened practice	Low-mid SES. Many patients of Greek background	Suburban practice Yet to go through accreditation	Community Health for AHPs	Fair IT deficiencies in new start clinic	Solo GP supportive	Aligned following GP / PN discussions	Fair – some GP/ PN communication difficulties	Positive Slow start until GP / PN discussions

^a^The government and regulatory aspects of each practice were shared given the similarity of the setting

**Table 2:** Percentage of charts with documentation of CVD risk indicators: baseline to 12 months post-intervention^f^

Practice ID	Active GPs/total FTE GPs^c^	Perceived practice engage-ment^d^	CVD risk^e^		Serum cholesterol		Blood sugar level		Blood pressure		Smoking status		Body Mass Index		Waist circumference		Alcohol consumption	
			Pre %	Post %	Pre %	Post %	Pre %	Post %	Pre %	Post %	Pre %	Post %	Pre %	Post %	Pre %	Post %	Pre %	Post %
A	3/3	Strong	70.4	77^b^	57.4	66.1^b^	1.6	3.8	79.2	79.8	95.4	95.9^a^	27.1	26.6	2.9	4.3	1.2	9.9
B	2/2	Strong	44.9	54.3^a^	43.1	48.1	42.9	45.4	64.6	72^b^	90.8	94.9^b^	12.6	19.7	3.9	4.8	11	12.5
C	4/13	Poor	39	44.7	54.5	N/A^g^	1.5	1.7	76.9	75.4	88.8	91.9	22.6	21	8.5	4.5	5.4	5
D	4/4	Poor ➔ Strong	32.4	45.2^a^	55.7	44.8	5.9	6.6	73.2	77.2^a^	73.8	85.7^b^	28	36.2^a^	3.2	12.7	18.3	31.2^a^
E	3/3	Strong	31.8	42.7^a^	43.7	57.1^b^	32.8	37.6	80.8	84.3^a^	93.2	94.5^a^	25.3	33.1^a^	6.3	9.8	38.9	37.9
F	9/12	Poor ➔ Strong	18.1	29.9^a^	51.8	56.7^a^	0.6	0.9	70.3	73.5^a^	35.5	49.4^b^	10.6	13.2	2.2	4.8	0	0
G	5/5	Poor	4.6	24.7^b^	8.9	47.6^b^	3.0	9.0	60.1	64	41.4	46.6	20.2	23.3	4.6	5.1	10.3	10.9
H	1/1	Strong	2.4	15.8	25.2	48.4^a^	5.6	9.6 ^a^	66.2	70.1^b^	35.8	46.1^a^	17.8	23.2	4.4	7.7	17.7	16.9

^a^indicates that post intervention there was an increase of between 10 and 19% in the documentation of the risk factors in those patient records without risk factor documentation at baseline. (also indicated by light shading)

^b^indicates that post intervention there was an increase of ≥20% in the documentation of the risk factor in those patient records without risk factor documentation at baseline. (also indicated by dark shading)

^c^The proportion of full-time equivalent GPs who consented to participate in the intervention related to the total number of FTE GPs in the practice

^d^Facilitator perception of practice engagement. The symbol ➔ implies transition of engagement over the course of the intervention

^e^Proportion of records with data sufficient to measure cardiovascular risk

^f^Targets for each indicator were cardiovascular risk: ≥40%, serum cholesterol: ≥50%, blood sugar: ≥50%, blood pressure: ≥80%, smoking status: ≥90%, body mass index ≥25% waist circumference: ≥10%, alcohol consumption: ≥40%

^g^Data unreliable for post intervention serum cholesterol calculation in Practice C

Our analysis of facilitator diaries found little difference between the frequency and content of facilitator contact with the practices. Facilitator activities followed core facilitation principles [18], were adapted to practice needs, used feedback of audit results, and frequently focussed on maximising the value of information technology systems.

At baseline, we found substantial variability in the prevalence of risk recording across the practices, in particular CVD risk (3.6–70.1%), smoking (35–95%), and BSL (0.6–42.9%). There was consistently low documentation of waist circumference and BMI. The best performing practices at baseline (A, B and E) were relatively small (around 3 FTE GPs), were cohesive, and were located in areas of mixed or affluent socio-economic status. Each involved non-GP staff in preventive activities, gave prevention responsibilities to PNs, and had preventive oriented clinical activities (i.e. a nurses’ room or a spare consulting room). Each was positive about the intervention.

The poorer performing practices at baseline (G and H) were in lower socioeconomic areas, served a multicultural clientele, and had opened in the previous 2 years. Practice G was a suburban, mid-sized practice with 5 GPs, several related by family, each of whom had been working separately until 2 years previously. Practice H was a solo practice with many non-English-speaking and aged clients.

At follow up, we considered improvements as being changes greater than or equal to 10% in the documentation of the risk factors in those patient records without documentation at baseline. Most practices showed such improvements in CVD risk factor and serum cholesterol documentation. All except practice C and G demonstrated a greater than 10% improvement in documentation of smoking status. Only one (practice H) showed any improvement in recording of BSL, and only one (practice D) made changes in the documentation of BMI, waist circumference, or alcohol consumption. The two practices with poorest performance at baseline made significant changes in three and four indicators respectively.

Interviews and diaries highlighted the fact that preventive activities were recorded in two ways in the clinical software. Some (such as cholesterol and (BSL) were measured through assessments of electronic entries into the relevant parts of the clinical software. By contrast, several measurements require clinicians to enter data into the clinical record after performing a clinical task (calculating BP, BMI or waist circumference, or enquiring about smoking and alcohol consumption). We found no quantitative evidence of “ceiling effects” across any of the original 15 intervention practices. For example, Practice A showed improvement despite very good performance at baseline. However, improvement was largely restricted to tasks requiring little clinician involvement.

### Interpretation of findings

Our narratives provide explanations for many of the patterns in practice responses to the intervention. It was clear that administrative and nursing staff were fundamental to change in practice performance. The role of the prevention coordinator (either PM or a PN) became critical in driving (or blocking) change in practices. PMs acting in the coordination role needed to change from being a “gatekeeper” to a “boundary spanner” between the front (administrative) and back (clinical) functions of the practice [19]. For example, rather than maintaining the divisions of labour between occupations, they needed to facilitate a collaborative change in activities, such as facilitating prevention related data collection by both front desk staff and clinicians. The frequent involvement of PNs in addressing prevention aligned with the increasing acknowledgement of their role in contemporary general practice primary care [20]. Further lessons emerged from: a practice that showed an unanticipated performance improvement; differences between two larger practices; and how practice software could generate quantitative results at odds with qualitative findings.

#### Unanticipated performance improvement

The facilitator spoke in detail of the challenges she faced in implementing change in Practice D, a medium sized practice of four general practitioners, three PNs and a PM. One of the GPs owned the practice and his wife was the PM (seen by the facilitator as a “gatekeeper, ‘controlling’ all information and decision making”). Many patients were non-English-speaking and/or overseas students. The practice was late to start the intervention. There was conflict between the lead PN and the PM and several of the GPs. Two of the three PNs supported the intervention, however the lead PN actively opposed its philosophy and content. She left some months after the intervention commenced.

As shown in Table 2, although its baseline preventive performance was modest in comparison with the other sites, at follow up Practice D was the only one that made major changes in all tasks requiring clinician input i.e. recording of smoking status, BMI, alcohol consumption, and waist circumference. Interviews showed how GPs valued the PEP audit and acknowledged the need to make changes in prevention work, prioritising the recording of smoking and alcohol consumption. Both the GPs and the facilitator felt that the resignation of the lead PN allowed the practice to develop a prevention focused team with two newly employed nurses. The practice met more frequently, spoke often of prevention and developed a working policy regarding staff roles, publicised preventive priorities in the waiting room, and used the nurses for risk assessment prior to the GP consultation. There was an increased focus on formal health assessments and a practice wide awareness of billing incentives for preventive activities. One GP commented, “*The facilitator assisted us to make decisions, about if we’re going to do any of this sort of quality medicine, we weren’t going to be able to do this quality type of thing with those particular staff members around.”*

#### Contrasting stories and different outcomes in practices of different size

Two of the practices (C and F) were large by Australian standards. Each had 13 GPs and had a lead individual in the practice that championed the intervention.

Practice C was owned by the lead GP and managed by his wife, the PM, who championed the PEP intervention, but was frustrated by the lack of involvement of the clinical staff as she suggested, *“I had lots of ideas, (but) virtually no one else wanted to participate.”* There seemed little impetus to change in the practice. GPs felt that even if there was a problem, there was little they could do to influence practice change. *GP3: “We already did a lot of prevention [and] didn’t really feel it was an issue that needed to be addressed. … [We lacked] true engagement, we did not own the process*.” The facilitator believed that the practice “*felt that they were doing pretty well and hence not much to focus on. [She saw it as the] practice that struggled most…”* No parameters showed any improvements following the intervention.

By contrast, practice F had a more broadly distributed ownership and decision-making structure than practice C. It was owned by four GPs who employed eight GP associates and three PNs. Nearly all clinicians in the practice were involved in PEP. At baseline, the practice had an ad-hoc approach to prevention, where individual clinicians rather than the practice as a whole took responsibility for preventive care. Although the facilitator perceived there to be only weak engagement with the intervention until late in the process, staff saw PEP as being able to generate whole of practice focus, where the PM was the main administrative driver of prevention and PN3 the driver of clinical activities. PN roles were expanded, use of both the electronic medical record and the clinical spaces changed and staff members assumed preventive roles. Practice F showed modest change in documentation of CVD risk and was the only practice to demonstrate changes in most behavioural variables.

#### Data and measurement surprises

Our case studies were able to explain anomalies in the data extracted from computerised medical records. For example, Practice G (one of the two poorest performers at baseline) relied on a busy PM as prevention leader, who was away for several months during the intervention. The facilitator found the practice challenging, busy, and wondered “*why they took on the project*” (IF). Only one GP and the PM seemed to demonstrate any more than passive support for the intervention. Nevertheless, the practice showed a fivefold increase in CVD risk recording, and a dramatic increase in the proportion of patients in the target age group who had their serum cholesterol recorded. There was no change greater than 10% for those preventive tasks reliant on active clinician documentation (BP, smoking, alcohol consumption, BMI and weight). Interviews revealed that these changes in recording followed the practice’s introduction of a new system for downloading pathology data into practice software.

## Discussion

Our study findings correspond with those of a series of facilitation intervention trials conducted within the US patient centred medical home, and raise questions concerning practice context, organisational readiness to change [21], and the measurement of the impact of facilitation intervention trials in primary care.

Stange and Glasgow’s recent characterisation of context in the patient centred medical home distinguished between inner and outer context. They saw *outer context* as representing government and broader organisational policies, community norms, system characteristics, and health system payment policies. *Inner context* represents a “practice’s ability to change”, and is represented by its *core* (personnel, processes and systems), its *adaptive reserve*, and its *relationship to the local health care neighbourhood*. The concept of adaptive reserve represents the degree of workforce capacity to participate in change projects [6]; leaders who embrace interventions; data driven improvement programs; and the functionality of the health care team [22].

Our sample’s outer context was typical of Australian fee for service family practice. Privately owned, each had similar models of care and broadly similar systems of reimbursement. Our assessment of intervention fidelity found no meaningful differences in how the facilitators worked with the practices.

Despite the fact that the external facilitators in this low intensity intervention made minimal explicit attempts to address the practice’s adaptive reserve, it was clear that this component of inner context was critical to changing performance.

There were some major changes in staff roles and, critically, the relationships between these staff. Practice D never really managed to get started with the intervention until a dysfunctional relationship between the PM and the lead PN was resolved by the latter’s departure. These findings correlate with work identifying the importance of leadership and inter-professional dynamics in fostering both teamwork related change [19, 23] and the response to facilitation in primary care [20].

### Practice size and context

Uncertainty continues to surround the influence of practice size on preventive outcomes [21]. Our findings also show some of the challenges implementing facilitation in large practices. The isolation, frustration and disengagement shown by practice C (where only 3 of 14 GPs participated) contrasted with the enthusiasm of the solo practice and showed how difficult it can be for champions to achieve practice change where intervention participation was isolated to a handful of practitioners. The experiences within practice C suggest that, despite the opportunity for interventions like PEP to affect more patients in larger practices, facilitation can be most effective where it is able to build understanding and intra-practice relationships. The trend is for increasing practice size throughout the western world, so if our findings are generalizable, it may become more challenging to facilitate change.

### The measurement of impact

This analysis highlighted the need for interventions such as PEP to consider the clinical routines underlying change in preventive performance. For example, a number of outcomes from the intervention i.e. assessment of BP, BMI, weight, alcohol, and cigarette consumption require clinical activity to be performed in an organised manner, documented, and then retrieved from the clinical record. The practices embracing the intervention demonstrated changes in most of these domains.

By contrast, at the time of our intervention, documentation of serum cholesterol was automatically imported from pathology providers into fields within each practice’s clinical records. The dramatic increase in the documentation of serum cholesterol in practice G was largely attributable to a change in this process of importing data rather than a change in the frequency of assessment.

### Limitations

This exploratory case study has clear limitations. We did not directly observe practice activities and while we oversaw data collection, the research assistants who conducted interviews were not part of the analytic team. However, we did carefully analyse by triangulating practice interviews, facilitator interviews, and facilitator diaries.

Observation itself can influence staff behaviour: interview data collection required a significant investment of time by practice staff and this may have generated a difference in intervention experience between study and non-study intervention practices.

Some studies have identified the importance of the individual attitudes aspects of health care professional engagement in primary prevention activities [24]. While our study included some data on this area, our focus was on engagement in quality improvement programs at a practice, rather than an individual level.

Unlike a number of other quality improvement interventions, the facilitators did not attempt to impact on team cohesion or other components of adaptive reserve. The overall impact of the intervention on outcomes, and the characteristics of the case studies may have been modified had we used team-training interventions as a foundation for other improvement efforts [25].

There were limitations to the assessment of the impact of the facilitation, especially related to the way in which assessments were recorded. Analysis of outcomes was reliant on practice-based data, availability of which depended upon practice staff processes for electronic filing.

## Conclusion

Facilitation interventions create the opportunity to tailor interventions to context. Our mixed methods study explored multiple layers of practice context and showed mechanisms underlying varying trajectories of change, the importance of relationships, and the importance of PNs and PMs.

The findings emphasise the importance of tailoring facilitation interventions to practice size, clinician engagement and, critically, the organisation of, and relationships between, the members of the practice team. Further research into practice facilitation will benefit from incorporating these elements of context, mechanisms, engagement, and relationships.

## Additional files

Additional file 1: PEP Practice Intervention - Study Outline. (DOCX 16 kb)

Additional file 2: Staff interview guide. (DOCX 29 kb)

Additional file 3: Facilitator interview guide. (DOCX 27 kb)

## Data Availability

The datasets generated and/or analysed during the current study are not publicly available as they contain identifiable patient information. Please contact the communicating author with any requests for access to the data-set.

## Abbreviations

BMI: Body mass index
BP: Blood pressure
BSL: Blood sugar level
CVD: Cardiovascular disease
FTE: Full time equivalent
PEP: Preventive evidence into practice
PM: Practice manager
PN: Practice nurse
