# Supplementary material for: Preventive Evidence into Practice: what factors matter in a facilitation intervention to prevent vascular disease in family practice?
Source: BMC Fam Pract. 2019 Aug 8;20:113. doi: 10.1186/s12875-019-0995-7 (PMC6688202; doi:10.1186/s12875-019-0995-7)
Supplement: Supplementary file 1 — PEP Practice Intervention - Study Outline. (DOCX 16 kb) [file 12875_2019_995_MOESM1_ESM.docx]

| Additional File 1  PEP Practice Intervention - Study Outline |
| --- |
| Rationale: The intervention was informed by a variety of theories and frameworks concerning organizational routines and patterns of work (1, 2), practice systems (Complexity Theory)(3-5), the way in which change is enacted and adapted in practices (Normalisation Process Theory)(6), a framework for organization of care activities (Chronic Care Model)(7), and P.R.A.C.T.I.C.E , a model for prevention in Australian general practice. (8, 9) A key task is to identify and enhance the supportive organisational infrastructure for the delivery of preventive care (8-11).  Aim: The Intervention was designed to improve assessment, recording, and management of vascular risk factors in general practice. Risk factors were the SNAPW risk factors: cardiovascular and diabetes risk, BP, lipids and renal disease.  Design: This study represented a “light-touch” facilitation intervention involving a workshop and face to face visits and telephone assistance from a practice facilitator.  Intervention details: Trained practice facilitators met with practice staff to assist in planning how preventive care could be provided. The discussion was informed by a baseline clinical audit of key vascular risk factors: absolute CVD risk, smoking status, alcohol intake, BP, BMI, waist circumference, and cholesterol and BSL. Then, participating general practitioners and PNs attended investigator led small group training (approximately 3-hour duration). Training used clinical scenarios and case studies to reflect on assessment, advice giving, goal setting, referral and follow-up of patients with abnormal risk factors.  Facilitators conducted three structured practice visits over 3 months following the small group training. Visits were focused on the implementation of a plan to improve the prevention of vascular disease in the practice population. The plan for each practice was required to include improving the assessment and recording of risk factors. Visits also provided patient education resources and information on referral services and programs within the local area. No specific prompts or templates for preventive care were used in the electronic record. Facilitators then provided telephone support to practices over 12 months.  Procedures/Materials: The intervention was structured around the 5A framework involving intervention by general practice staff as well as appropriate referral to other services and programs. Central measures were developed through a clinical audit of eight key vascular risk factors: absolute CVD risk, smoking status, alcohol intake, BP, BMI, waist circumference, and cholesterol and BSL. Recording targets were set for these measures. Practices received written feedback of a clinical record audit of recording and levels of behavioral and physiological risk factors (BMI, waist circumference, systolic BP, alcohol intake, smoking status, lipids, fasting BSL and absolute CVD risk) for chronic vascular disease from the electronic medical record of each practice. These were compared with Australian evidence-based guidelines and standards.  Tailoring: Practice goals to improve other aspects of vascular preventive care were tailored for each practice based on need and the priority of participants. |

1. Becker MC, Lazaric N, Nelson RR, Winter SG. Applying organizational routines in understanding organizational change. Industrial and Corporate Change. 2005;14(5):775-91.

2. Becker MC. Organizational routines: a review of the literature. Industrial and Corporate Change. 2004;13(4):643-78.

3. Miller WL, Crabtree BF, Nutting PA, Stange KC, Jaen CR. Primary Care Practice Development: A Relationship-Centered Approach. The Annals of Family Medicine. 2010;8(Suppl 1):S68-S79.

4. Stroebel CK, McDaniel RR, Crabtree BF, Miller WL, Nutting PA, Stange KC. How Complexity Science Can Inform a Reflective Process for Improvement in Primary Care Practices. Jt Com J Qual Patient Saf. 2005;31(8):438-46.

5. Plsek PE, Greenhalgh T. Complexity science: The challenge of complexity in health care. BMJ. 2001;323(7313):625-8.

6. Gunn JM, Palmer VJ, Dowrick CF, Herrman HE, Griffiths FE, Kokanovic R, et al. Embedding effective depression care: using theory for primary care organisational and systems change. Implementation science 2010;5:62.

7. Bodenheimer T, Wagner EH, Grumbach K. Improving primary care for patients with chronic illness. JAMA. 2002;288(14):1775-9.

8. Litt JC. Exploration of the delivery of prevention in the general practice setting [PhD Thesis]. Adelaide: Flinders; 2007.

9. Royal Australian College of General Practitioners. Putting Prevention into Practice. Guidelines for the implementation of prevention in the general practice setting. 2nd ed. Melbourne: RACGP; 2006. 94 p.

10. Stetler CB, McQueen L, Demakis J, Mittman B. An organizational framework and strategic implementation for system-level change to enhance research-based practice: QUERI Series. Implement Sci. 2008;3:30.

11. Solberg LI. Improving medical practice: a conceptual framework. Ann Fam Med. 2007;5(3):251-6.
