# Supplementary material for: Preventive Evidence into Practice: what factors matter in a facilitation intervention to prevent vascular disease in family practice?
Source: BMC Fam Pract. 2019 Aug 8;20:113. doi: 10.1186/s12875-019-0995-7 (PMC6688202; doi:10.1186/s12875-019-0995-7)
Supplement: Supplementary file 2 — Staff interview guide. (DOCX 29 kb) [file 12875_2019_995_MOESM2_ESM.docx]

**Additional material 2 – Staff interview guide**


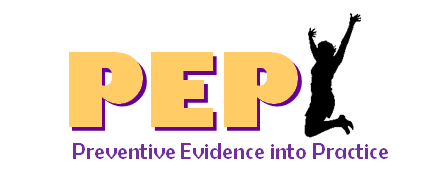


Interview Guide:

General Practitioner, practice nurse, practice manager

Introduce the purpose of the interviews**:** *This interview is to help us to understand the practices approach to improving preventive care for vascular conditions - especially the detection and management of the SNAPW lifestyle risk factors, hypertension, dyslipidaemia and pre-diabetes – and the impact of the facilitation provided by the project to the practice.*

**EARLY IN THE INTERVENTION PROCESS**

*(prompts in italics)*

1. How would you say your practice deals with preventive care for patients? What does the practice try to do?
2. Sometimes practices say that there are differences in the way that different members of the practice deal with prevention? Is that the case in your practice? Why is this?
3. Have you changed or tried to change anything in the way you provide preventive care during the past 12months prior to the project? (e.g. patient education, health assessments)

***If no skip to question 4!***

***If yes****: Could you tell me about this change? How did you make this change part of your routine practice? What did you have to do to achieve this?*

- - *How did this go? Did it seem to make any differences in how care was delivered? How was this for the staff in the practice? For the patients?*

1. There are a lot of variations in how practices deliver preventive care. What factors influence how effectively your practice is able to provide preventive care?
   - *Patient factors*
   - *Own knowledge or confidence/ own experience / belief in success of interventions*
   - *Your practice (staff organisation)*
   - *Availability of resources outside the practice (ie allied health, community health…)*
   - *The organisation and funding of health care*
   - *Other… Do others in the practice have different views…*
2. You were provided with an audit report on preventive care. How did you find the audit report?
   - *Problems with the audit*
   - What issues or priorities did you identify from this?
3. This project has an educational meeting and a series of visits from a facilitator. Have you experienced this sort of a program in the past about preventive care. How do you feel about the visits?
4. Demographic details:
   - Gender
   - Past training – special interests
   - Past practice experience with facilitation (i.e. collaboratives)
   - Years in general practice

**POST INTERVENTION INTERVIEW QUESTIONS**

(prompts in italics)

1. We are interested in asking about the changes that have come in the practice following the end of this project. We were hoping to address issues around prevention of cardiovascular disease in general practice. Tell me the story of your involvement with the project (attending workshop, facilitator, audit other…)
2. What has changed in how the practice manages preventive care since the intervention?
   - *What people do / how they do it / other changes (meetings, staff roles, time spent, patient perceptions of all of this…*
   - *Why do you think that has happened?*
3. We are interested in learning about what types of support did the facilitator provide (examples: setting goals for your personal practice, giving feedback, etc)?
   - *Which of these were most helpful?*
   - *Which of these were least helpful?*
4. What types of support did the facilitator who visited the practice provide (eg setting goals for your practice, giving feedback, providing resources
   - *Did it actually make a difference? If yes, what would make the facilitation process work optimally? What supports would you need for success?*
   - *If it didn’t make a difference, why not?*
5. How sufficient was the support you received in planning improvements to preventive care?
   - *What else was required?*
6. What would you recommend we change/keep the same if we were to try this again? Do you see potential for further use of this facilitation process in your practice and with others?
7. Any other comments
8. Demographic details:
   - Gender
   - Past training – special interests
   - Years in general practice
   - Years in this practice
