# Supplementary material for: Preventive Evidence into Practice: what factors matter in a facilitation intervention to prevent vascular disease in family practice?
Source: BMC Fam Pract. 2019 Aug 8;20:113. doi: 10.1186/s12875-019-0995-7 (PMC6688202; doi:10.1186/s12875-019-0995-7)
Supplement: Supplementary file 3 — Facilitator interview guide. (DOCX 27 kb) [file 12875_2019_995_MOESM3_ESM.docx]

**Additional Material 3 Facilitator interview guide**


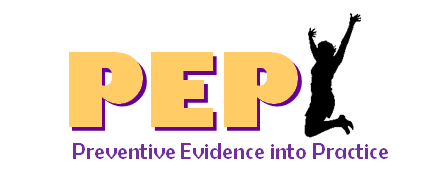


Interview Guide: Facilitator

*1) Characterize the Facilitator’s experiences with implementing components of the intervention and*

*2) Explore their perceptions of critical practice and practitioner factors impacting the fidelity of the intervention.*

Begin with an icebreaker:

- *Thanks for agreeing to be interviewed. We are interested in speaking with you about your experiences in the implementation of the PEP program of work. I haven’t been involved with any of the decision making about the project and am in fact learning about it over time….*

##### Could you tell me about how you came to be involved?

- What was your first contact with the project? What happened then (getting an idea of timelines)
- Had you done similar work in the past? Can you tell me about this?

###### How would you describe what you were trying to do in this project?

- What you were asked to do in the PEP project? (ie recruitment, practice contact, follow up).
- How did you find the experience of recruitment?
- How did you construct your work within the practices?
  - What sort of contacts did you have… *tel calls/visits/other.*
  - What use did you make of the pre-intervention audit?
  - The group meeting with the investigator?
  - Other tools?

###### I’d now like to speak more specifically about the practices…How many practices did you cover?.

###### Can you tell me a little about each practice?

- - *Location/Size/practice nurse/culture and leadership/ funding model (bulk billed etc)*
- In which practice did things seem to work best? Why do you think that was? What were the factors that made a difference?
  - *Probe on: model of care (traditional GP centred, or collaborative inter-professional); nurses’ roles, presence of practice “champions”,); practice ownership; practice size, practice culture and leadership, clinical governance processes; funding resources; practice physical spaces; medical neighbourhood connections to other practices, ML etc; connections to professional associations*

#### What does success look like to you? How do you know when you have got there?

- What practice was the most challenging? Why do you think that was the case?
- What practice was the most accepting? Why do you think that was the case?

I would now like to ask about some of the components of the program

- What were the practices able to do in implementing the SOC/5As (point to document and discuss).
- Which activities do you feel were most helpful / most challenging to participants.
  - *Probe: culturally appropriate educational resources, guide to use RACGP PrimaryCare Sidebar, local referral directory, ML run training sessions, Intervention Practice Visits, follow-up telephone calls, troubleshooting telephone calls*

Now I would like to ask about your preparation for the program

- How well prepared did you feel before going into the practices?
  - *the initial training day / the manuals / investigator assistance / support from the other PEP team members (ie the teleconferences) and other measures?*
  - *(If they respond negatively) What could have been done differently to improve the preparation?*
- How well supported did you feel throughout the intervention?
  - *Probe: other team members, investigators teleconferences etc?*
  - *(If they respond negatively) What could have been done differently to improve the preparation and support?*

We are nearly at the end. I would like to ask you to reflect on your whole experience with the program.

- Think back to when you first joined the program, please tell me what transpired to be different from what you expected.
- If you were to implement this type of program again, what would you change?
- How well do you think you went in the intervention?
- Will you be able to take any of the experience from this work into future work (e.g. with the Medicare Local)?

And now a few things about yourself:

- Education/training/current work

Is there anything that you would like to add that could help me in understanding your work and that of the program?

Do you have any questions?
